# Notes of a Visit to the Public Lunatic Asylums of Scotland

**Published:** 1856-04-01

**Authors:** John Webster

**Affiliations:** Physician to the Scottish Hospital, &c.


### Art. III.—NOTES OF A VISIT TO THE PUBLIC LUNATIC ASYLUMS OF SCOTLAND.

BY JOHN WEBSTER, M.D., F.R.S., AND F.R.C.P.,

*Physician to the Scottish Hospital, &c.*

(*Continued from page* 66.)

### Murray's Royal Asylum.

This institution for the insane, founded in 1827, is situated near Perth, upon the north-western declivity of the picturesque hill of Kinoull, having a magnificent prospect over the basin of the river Tay, with the Grampian Hills on one side, and those of Strathearn on the opposite. Being constructed on a kind of slope, the lower story windows of the building are nearly on a level with the adjacent ground in front, whereby, this part of the structure is rendered rather dark, somewhat damp, and consequently not so airy or salubrious as, the upper portion. In this respect, the asylum partly resembles that of Colney Hatch, where the same objectionable peculiarity prevails. Having been erected prior to the introduction of many modern improvements, which science and more extended experience have introduced into recently-built asylums, several defects might be fairly pointed out by hypercritical observers, but which need not be now mentioned; as altogether, this establishment deserves much approval in reference to its general management, and especially, with regard to the treatment of patients therein confined.

One great defect characterizes this asylum,—namely, its present scanty supply of water; and, what is still more regrettable, there is very little prospect of this deficiency, in so necessary an element in every institution for the insane ever admitting an effectual remedy. Being placed so much above the neighbouring river Tay's level, and the city of Perth, any supply from that locality can never likely be conveyed to this high position. Farther, as adjoining landlords have objected to water being brought from sources on their property, the institution has to depend chiefly upon wells sunk within its own precincts, aided by rain reservoirs. The gardens attached are extensive, well arranged, highly cultivated, and appeared exceedingly productive. They produce plenty of fruit and vegetables for the establishment, besides being, along with the adjoining fields—belonging to the asylum—of great use to the patients, as such appendages supply ample means for giving inmates employment, as also affording amusement and healthy recreation.

Similar to every public institution for the insane throughout Scotland, the Perth asylum receives both pauper and private patients. Of the latter description, there were 50 inmates at the time of my inspection, who paid from 80*l.* to 250*l.* per annum, board and lodging included. The pauper, or lowest class of patients, pay eight shillings per week, which is certainly very moderate, considering the recent high price of provisions. Taken in the aggregate, the total lunatic inmates, on the day of my visit to this asylum, amounted to 141 individuals, of whom 77 were males and 64 females: hence giving, as at Glasgow, more of the former sex than the latter. Amongst the above, only 2 men laboured under general paralysis; 2 males and 6 females were dirty patients; and 1 male and 2 females suffered from epilepsy; the other cases being mostly of a chronic description. No patient was then under bodily restraint, excepting two males, who had strong leather gloves temporarily upon both hands, to prevent them destroying their clothes. However, one of these persons had been only recently brought to the institution, in a violent state of excitement, which seemed not yet abated. One female lunatic also was placed in an open court-yard, to avoid all annoyance to other patients through her violence, but none were actually in seclusion; and the strait-waistcoat appeared unknown, or had become a matter of history.

During last year, 36 new insane patients were admitted, of whom 23 were males and 13 females; 6 males and 11 females were discharged cured, whilst 7 male and 8 female lunatics died: 7 of the above deaths having been caused by cholera, which visited the establishment last season as an epidemic. Respecting the form of insanity in the cases admitted, it appears 8 were examples of mania, 8 melancholia, 9 monomania, 7 dementia, 2

general paralysis, and 2 dipsomania. In 8 of the above patients, a suicidal propensity exhibited itself more or less strongly; in 10, the insanity assumed a religious type; in 2 a homicidal and mischievous tendency existed, whilst in 2 examples dementia was complicated with epilepsy. Besides the 7 deaths by cholera, and 3 from choleraic diarrhœa, 1 case terminated fatally from organic disease of the heart, 1 by apoplexy, 1 in consequence of typhoid broncho-pneumonia, and 2 deaths occurred through senile exhaustion.

Occupying the patients constitutes a prominent feature at this asylum. Consequently, in the female work-room, millinery and needlework is an ordinary employment, whereby a large quantity of the clothing required for patients is both made and mended. The laundry likewise supplies regular occupation to various female patients, as also the kitchen and ordinary household duties. In this way a proportion of convalescents, or of those inmates labouring under milder forms of mental disease, materially promote the comfort of their less fortunate companions in affliction, whilst they also assist essentially in maintaining the establishment's efficiency. Shoemaking, carpentry, besides outdoor work in the garden or grounds, further afford daily occupation to numerous male patients, the latter manual labour being both useful and healthy for the insane. Amongst persons of education, especially in the higher classes, it is not always so easy to engage such residents in any kind of physical employment. Still, on the male side, various inmates are employed as book-keepers, drawing ornamental designs, in making fishing-tackle, or by playing on musical instruments. Some amuse themselves at cards, bagatelle, chess, or billiards; whilst others again frequent the bowling-green, in order to enjoy the healthful exercise thus afforded. Amongst lady patients, playing on the piano supplies a constant source of pleasure to several, besides those who are occupied in needlework, reading, or in writing. Some spin, or manufacture wax-flowers and ornamental knick-knacks. Hence, but few remain idle, and so avoid feeding on their own morbid illusory sensations, which often prove injurious.

Irrespective of the zealous attention paid at this asylum to occupying the inmates, whether pauper or otherwise, in manual or bodily labour, recreating them in various ways, besides promoting their intellectual culture, wherever possible, now constitute prominent features in its general management. Upon both these important questions, a better exposition cannot be conveyed to readers, than extracts from the recent annual report, which states:—

"The introduction of recreations among the insane can no longer be regarded as an experiment; their success has been fully established by the experience of the best asylums in this country, on the Continent, and in America. They are not to be looked upon as mere transient gratifications, or as frivolous, and tending to dissipate, degrade, or pervert the mental energies or moral feelings of the insane. Our own experience of their curative value has been most encouraging. We have frequently observed the first symptoms of improvement in the form of a smile, a laugh, a critique, or a mark of applause, at a ball or a concert; we have seen the most fatuous, apathetic, and indolent patient—the melancholic and suicidal, as well as the proud monomaniac—delighted with some lively or familiar music, or dancing with the greatest vivacity at the weekly balls. Five concerts were given during the winter, in presence of between 40 and 70 patients. The performances included every variety of vocal and instrumental music, and the performers—composed of a mixture of patients and officers—numbered from 10 to 20. No audience could have been more attentive, delighted, or grateful; none could have behaved with greater propriety."

Pic-nic excursions to the neighbouring beautiful localities, *fêtes champêtres*, athletic games, such as cricket, bowls, quoits, or football, with promenades in the grounds, and pedestrian tours to various places of interest in the vicinity of Perth, are frequently made; whilst parties have likewise been sent into that city, to attend various public concerts and exhibitions, as also the annual races.

Respecting the education and intellectual culture of lunatic inmates at this establishment, besides conversaziones held occasionally, during the earlier months of winter, it is further stated in the document already quoted, that—

"During the later winter months, a course of lectures on Economic Botany, or the applications of botany to common life, was delivered on the Saturday evenings to an average audience of thirty persons, belonging chiefly to the higher classes of patients. By means of presenting objects of natural history and scientific information under an attractive guise, we have found the insane become accurate observers, apt students, and frequently acute reasoners. The remarks made at the conversaziones following each lecture, showed that the patients present had not been mere passive or quiet listeners, but that their minds had laid hold of many facts which not only served as nuclei for present reflection, but formed solid increments to their stock of knowledge. The gradual acquisition of scientific information by the insane not only tends to lead to habits of correct observation, stimulate to the study of the good and beautiful in nature, furnish the mind with the highest and purest subject-matter for contemplation, and attract attention from the gross and degrading pleasures of the world, but it may become of infinite service to them in after life, when they recover their mental equilibrium, and resume their places in society. As an immediate and gratifying result of these scientific meetings, four lectures were delivered by two patients to the same audience, the subjects being respectively 'The Beauties of Nature,'

'Antiquities of Perth,' 'Comicalities,' and 'Superstitions of the Highlands.' These lectures were treated in such a way as to render them popular and attractive; and a cordial vote of thanks was awarded by the audience in approbation of the useful though unpretending labours of their authors. The preparation of such discourses is not only valuable, by diverting attention from morbid fancies and unpleasant associations; but the amount of reading necessarily involved cannot fail to leave a lasting and useful impression."

Statements like these are exceedingly gratifying; and as showing how the system pursued at this institution is proposed to be carried out, during the ensuing season, an account of the classes and lectures intended for inmates, throughout the winter of 1855-56, is now appended, viz.;—

I.—Theory and Practice of Vocal Music, under the direction of Herr Boos, Professor of Music, from Bonn-on-the-Rhine.
Thursdays, 5·30 p.m.

II.—Sub-Class for the Practice of Vocal Music, under Mr. Williams.
Saturdays, 6·30 p.m.

III.—Practice of Psalmody, under Miss Blake.
Wednesdays, 6.30 p.m.

IV.—Dancing and Deportment, under Mr. Guthrie.
Mondays, 6·30 p.m.

V.—Reading, Writing, and Arithmetic, under Miss Norman and Mr. Williams.
Fridays, 6·30 p.m.

VI.—Religious Instruction, under Miss Norman.
Sundays, 6·30 p.m.

VII.—Lectures. The Course of Lectures on "Economic Botany" commenced last winter, will be continued.

Arrangements have also been made for the delivery of the following:—

1. Galvanism; its phenomena and economical applications, illustrated by the electro-magnetic apparatus.

2. The Blood; its composition and uses, illustrated by the microscope and by diagrams.

3. Time; its proper occupation and uses, illustrated by anecdotes of the "pursuit of knowledge under difficulties."

4. Jacobite Minstrelsy, illustrated by Jacobite melody, vocal and instrumental.

The Rev. Mr. Murdoch, of Kinnoull, Dr. Stirling, of Perth, and other Gentlemen, have kindly promised their assistance in this department.

Besides these laudable exertions to occupy and cultivate the mental faculties of lunatics whilst under treatment at this asylum, their amusement likewise constitutes a prominent peculiarity; hence, balls and musical entertainments are of no unfrequent occurrence. Thus, a grand concert was recently given, the following being a copy of the printed

PROGRAMME.

PART I.

| | | |
|---|---|---|
| 1. | Opening Address | Manager. |
| 2. | Piano and Violin—"Bo-peep Quadrilles" | Miss Blake. Mr. Gowenlock. |
| 3. | Trio—"Canadian Boat Song" | Mad. Monti. Madl. Marie. Mr. Gowenlock. |
| 4. | Song—"Oh! Native Scenes" | Miss Blake. |
| 5. | Recitation—"The Well of St. Keyne" | Miss Norman. |
| 6. | Comic Song—"The Whale" | M. Guillaume. |
| 7. | Song—"My ain dear Nell" | Madl. Marie. |
| 8. | Trio—"Taste Life's Glad Moments" | Misses & Mr. Gowenlock. |
| 9. | Song—"The Soldier's Return" | Mad. Rothe. |
| 10. | Violin—Selection of Scotch Airs | Mr. Gowenlock. |

DURING THE INTERVAL A

PAS DE DEUX . . . . . . . MASTERS GOWENLOCK.

PART II.

| | | |
|---|---|---|
| 11. | Piano—Quadrille Music | Mad. Catherina. |
| 12. | Duet—"All's Well" | Miss Blake and Mr. Gowenlock. |
| 13. | Comic Song—"The Charming Woman" | Miss Norman. |
| 14. | Recitation—"The Chestnut Horse" | Mad. Joseph. |
| 15. | Trio—"Bonnie Wood o' Craigie Lea" | Misses & Mr. Gowenlock. |
| 16. | Duet—"The Araby Maid | Madl. Marie. M. de St. Pierre. |
| 17. | Song—"Rowan Tree" | Madl. Marie. |
| 18. | Aria from "La Sonnambula," "Still so gently o'er me stealing" | Miss Blake. |
| 19. | Comic Song—"Lilly Baker" | M. Guillaume. |
| 20. | Chorus—"Auld Langsyne" | Corps Musical. |

Doors open at half-past Six, Performance to begin at Seven precisely.

CONDUCTOR, HERR WISTOWSKI.—PIANIST, MADAM ANGELICA.

Contrasting these exhibitions with the harsh system formerly pursued throughout many parts of Europe, even until very recent times, and which have not yet been altogether laid aside in some countries I might name, Scotland seems now to take the lead in this onward movement. Of course, the genius and natural bent of its people render such occupations as have now been portrayed more congenial than would perhaps be found, for instance, in England, where music and dancing possess, by no means, the same attractions as north of the Tweed. This opinion may be exemplified by the following quotation from a recent report of the

Wilts Asylum, written by the physician, Dr. Thurnam, which says: "Dancing, we believe, seldom forms a part of the ordinary amusements of the reputable poor of Wiltshire, and the medical superintendent entertains considerable doubt as to the propriety of its formal introduction into asylums for the poor, at least in this part of England." Such opinions, certainly, do not apply to Scotland, or even to Metropolitan asylums, and some institutions in Ireland, where the case becomes no doubt often different. At Bethlem Hospital, and the Colney Hatch Asylum, for instance, musical parties and evening entertainments have occasionally been given to the lunatic inmates; but no English insane establishment appears yet to have surpassed that of Perth, in the above peculiar features.

The authorities of Murray's Asylum being deeply impressed with the conviction that, no more powerful moral medicines, wherewith to "minister to a mind diseased," can be advantageously employed than occupation, recreation, and education, they have consequently endeavoured to extend and vary the modes formerly in use, of employing, amusing, and instructing the patients committed to their charge, according to the capacities or acquirements of different inmates. In these praiseworthy endeavours, they have been most zealously and efficiently seconded by Dr. Lindsay, the present resident medical officer and superintendent, who was recently attached to the Royal Crichton Asylum, Dumfries: in which psychological school, so well known by the philanthropic labours of its eminent physician, Dr. Browne, already several able medical superintendents of public lunatic establishments have been educated, and afterwards translated to fill superior situations, in other similar institutions. To carry out these objects, besides a good library, this asylum possesses a considerable collection of curiosities, with various periodicals and newspapers; whilst the study of the beautiful, and a familiarity with Nature's works, is further fostered by introducing various flowering plants into the different galleries. These become not only pleasing to the eye, but otherwise prove useful, by encouraging the formation of bouquets among ladies: which taste is further aided by cultivating roses and annual flowers in the garden or ornamental *parterres.*

The professional attendants of this Asylum are Dr. Malcom, an eminent physician residing in Perth, and Dr. Lindsay, the resident medical superintendent: of whom I would here briefly say, they merit much commendation, both being zealous and efficient officers in their respective departments.

### Dundee Royal Asylum.

This public establishment for lunatics, which has, since its foundation forty-three years ago, enjoyed a high reputation throughout

Scotland, is situated in the immediate vicinity of the town whose name it bears. The ground occupied slopes toward the river Tay, over and beyond which its inmates possess an extensive prospect, as also along the opposite coast of Fife, extending up even to Perthshire. In consequence, however, of the increasing size of the flourishing commercial sea-port—now a near neighbour—this formerly excellent, open, and unobstructed situation has, of late years, been greatly deteriorated: whereby, several fine views from the windows and court-yards are much spoiled by the roofs of not very distant houses or factories, and even by various tall smoking chimneys which intervene. Attached to the Asylum there is an excellent garden, which, with the adjoining fields, also under cultivation, comprise altogether about thirteen acres. Since 1812, when the Dundee Asylum was first founded, many extensive and important additions have been made at various times to the building and precincts. At its first opening, accommodation was provided only for forty patients; now, it could receive nearly six times that number. This enlargement has enabled the authorities to dispense with various antiquated and inconvenient appliances, so as thereby to satisfy many essential requirements for promoting the well-being, recreation, and recovery of lunatic inmates under treatment.

When I visited this institution last September, the total insane patients amounted to 212; 94 being males and 113 females. These were divided into two classes, pauper and private; the latter category then amounting to 36 persons, whose board varied from one to three guineas per week. Amongst the aggregate patients, 7 were epileptic males; but no female inmate laboured under that severe form of mental disease. Those classed as dirty patients comprised 15 cases, of whom 8 were males and 7 females. The physical health of all appeared so good, that only one female remained sick in bed; and not a single individual was then in confinement, seclusion, or subjected to restraint of any description whatever. In short, the discipline, general appearance of the establishment, quietude, and outward comfort of the patients seemed altogether satisfactory; and I have seldom been more gratified when perambulating any institution for the insane, than on the occasion now mentioned. Having been accompanied by my honourable friend Mr. Duncan, M.P. for the borough, during this inspection, that gentleman became also cognizant of the facts here detailed, and will doubtless verify the general statements I have promulgated.

During the past year 51 new patients, including readmissions, were received, 26 being males and 25 females; 12 males and 9 females were discharged convalescent, and 11 died, of whom 6 were male, and 5 female inmates. If the latter numbers be calculated according to the admissions, the ratio of cures hence amounted to 41 per cent.; the deaths being 21·57 per hundred. The forms of disease manifested, by new cases, were chiefly mania and dementia: there being 18 examples of the former, and 19 of the latter; whilst 8 instances were classed as monomania, and 6 as melancholia. Respecting the causes assigned, drunkenness, like the fact noticed at Glasgow, appeared the chief influence—9 victims being ascribed to that calamitous propensity; 7 arose from disappointment, and 3 from grief. Further, 2 were puerperal cases; 2 seemed produced by fright; 1 female became mad through jealousy; another by religious excitement; but in several the cause was unknown: whereas in 10, hereditary predisposition was ascertained. Again, respecting the immediate causes of death reported in the 11 fatal cases: 4 arose from general paralysis, of whom 3 were male patients; 2 were occasioned by bronchitis; 2 by marasmus; 1 from pneumonia; 1 through cardiac disease; and the eleventh was ascertained to be gastro-interitis.

Occupations of different kinds, adapted to the previous employments, general character of inmates, and the nature of their mental condition—all requisites of essential importance—seemed assiduously promoted. Various patients, both male and female, appeared occupied at the weaving-loom, or in winding yarn—these being common employments in this district of Scotland. Some were engaged in shoe-making, mending or making clothes, and so forth; whilst others laboured in the garden. The kitchen, scullery, and laundry likewise afforded ample scope for beneficial occupation. One patient may be mentioned as an apt illustration, who believed herself pursued, at night, by the devil, and bands of warlocks, or witches. This poor girl offered her services to the matron, as kitchen-maid, and continued to work diligently; presenting herself at each succeeding hiring-term, to ascertain whether she would be re-engaged: but appearing all the while both happy and contented with the treatment received. Many other insane residents, whom it is superfluous to specify on this occasion, were supplied with varied but appropriate means of gratification, and opportunities for activity, if considered suited to their different conditions. Nor were inmates always debarred personal intercourse with the external world, or even from occasionally mixing in general society. The town and neighbouring country being occasionally visited by patients, for various purposes, both on foot or in carriages. Excursions were also sometimes made to a distance. For instance, during last autumn a lady patient, accompanied by the matron, with an attendant, travelled to Balmoral, and saw the queen in Crathie Church. Afterwards, she visited some fine Highland scenery, and then returned back to the asylum; her excursion having proved both beneficial and satisfactory.

Amusements likewise are judiciously provided to gratify and benefit the lunatic inmates. Frequent dancing parties take place; and even a customary thrice-a-week ball is held in the large flower-garden, where spectators and musicians occupy an orchestra, raised upon a small mound, surmounted by a flag; whilst ample opportunity is thus given to others, for indulging in merriment, and innocent social tendencies. On the occasion of our perambulating the various dormitories and court-yards of this establishment, a male patient, who was an excellent performer on the violin, accompanied us throughout. This amateur musician played exhilarating Scottish tunes, whilst we were visiting the wards, which apparently caused much delight to the numerous gratified auditors then encountered. The fiddler seemed a welcome visitor amongst both male and female inmates wherever he went; and I could not but observe the pleasure thereby occasioned to many of these mentally-afflicted fellow-creatures. Having met, in one of the gardens, a party of female lunatics enjoying their customary promenade, they immediately formed themselves into a kind of squadron behind our "*fanatico per la musica*," who then marched at their head, several singing, and even some dancing in the rear. Altogether, the scene was a very pleasing spectacle; their enthusiastic musical leader being an universal favourite, and caused quite a sensation when making his appearance.

Although lunatic asylums seem frequently the abodes of melancholy, and residents may sometimes have but few gratifications, certainly, on the occasion now described, hilarity and pleasure beamed in many countenances. Besides which, I may add, the general aspect of the asylum, and other outward marks, unequivocally manifested the physical comfort of the inmates. On these points there was no mistake; and, I must say, much credit is due to Dr. Wingett, the resident medical superintendent, as also the matron, Mrs. Wingett, for the systematic mode they pursue in reference to the amusement, as also recreation of patients placed under their charge and professional treatment.

A new church is now being erected, in a pleasant part of the adjoining grounds. This building has much the appearance of an ordinary parish kirk, in the light Norman style; and as there will be a continuous covered way connecting it with the asylum, patients can thus always arrive dry-shod during unfavourable weather: whilst, if fine, a walk through the garden-grounds must prove agreeable, besides being very like frequenting an ordinary country church for public worship. Within this precinct no prison-like separation of the sexes, it was said, will be made in the interior arrangements: which sometimes prevails elsewhere, so as thereby to remind spectators very much of some model dungeon-chapels, wherein a large board jealously separates males from ever seeing the faces of any female worshippers, there also assembled: and *vice versâ*. Such minute distinctions may suit criminals, but amongst lunatics, the system becomes as absurd as it is injurious. Hence, in several modern and well-regulated asylums, this plan is generally abolished, the object being to render church attendance as near as possible similar to that pursued in ordinary places of worship, although the females are usually placed on one side, while male patients occupy the opposite.

Amongst individual cases recently under treatment, I would quote the following brief remarks from a late report of this institution, to illustrate its proceedings, particularly as the facts are also otherwise interesting:—

"Thus, one man who recovered during the year, passed his time—which was all divided into portions devoted to occupations of his own choice—in painting in water-colours, in reading the German poets, conning the newspapers, and smoking cigars in the garden. He improved rapidly, and when questioned as to his wants and welfare, was accustomed to reply that he had everything he required or wished for; that he felt no more restraint than if he were in an ordinary hotel; and that he had worked himself in a state of good health by his laborious artistic exercises. Another devotes most of his time to the rearing of poultry; he cannot be diverted from this to any more elevated employ; and living like a Rosicrucian philosopher upon the golden delusion that he is above death or corruption, and that his body will continue to flourish through all eternity in its present vigour, he seems to experience the highest happiness. A third passes much of his time in acting as the amanuensis of the medical superintendent, and indulging the belief that he has the regulation of the weather under his control, is the tutelar divinity of farmers, and can make the sun to shine, or the rain to fall, at his bidding; he passes a very industrious life, in the assurance that he is the most important and responsible man upon the face of the earth."

It may be further mentioned that, among the individuals received into the institution during the past year, one imagined he had a Divine commission for fire-raising and assaulting individuals. Another believed himself tempted by two imaginary personages to kill his wife. A female made an attempt to strangle her husband, while he was asleep. A fourth, during her frenzy, was happily saved by friends from destroying her children. And lastly, a young female, in whom the incursion of insanity was marked by her plundering and embezzling the property of her friends. These facts indicate the fearful tendencies and tyrannous controlling power of some forms of mental alienation, which are not unfrequently met with in asylums for the insane.

However much many arrangements are worthy of commendation, nevertheless, one feature in the management of this public asylum is highly objectionable,—namely, the regulation requiring a fee for the physician, to be paid by the several classes of patients, excepting on account of paupers sent by parishes. The sum varies from *half-a-guinea* to four guineas, which is repeated on the dismissal or death of an inmate, after six and within twelve months. If, however, any patient remains longer than one year, such payments are only demanded at the end of every successive year of residence. In all public institutions, the salaries of medical officers ought to be liberal, but fixed; there should be no distinction of classes, in reference to professional remuneration; nor must any premium be even in appearance held out, to give more attention towards one inmate than another; nay, of at all making it the interest of any person in such establishments, whether medical attendants or otherwise, to admit or retain longer than may be necessary, within an asylum, patients who prove, compared with others, in a higher ratio, remunerative. Physicians ought never to be subjected to similar regulations, as they thus become placed in an unprofessional position. Consequently, the above rules—copied from the "Rates of Board" appended to the last Report of the Dundee Royal Asylum—should be repealed, and expunged from similar future documents issued by its executive.

Like the Murray Institution near Perth, this establishment has only one resident medical officer, — viz., Dr. Wingett, who is superintendent. There is likewise a physician, but he resides in Dundee. During many years, this office was filled by Dr. Patrick Nimmo, long known as an eminent practitioner throughout the country. Death having recently deprived the institution of his valued services and great experience, Dr. Robert Cocks has been appointed as successor. Still, in this asylum, containing about 210 lunatic inmates, on an average, besides its numerous attendants, Dr. Wingett should have, if not a resident assistant, at least be allowed one or two house-pupils or "internes," as in France, to aid in minor duties, and to be available should any emergency occur, during his temporary absence. The advantage of young members of the profession residing in similar establishments, seems so obvious as scarcely to require any argument. Not only do such officials prove of much assistance to the responsible medical attendant, but being so placed, "internes" have ample opportunities for studying mental diseases, their nature and treatment, so as thus to prepare them in the best school for afterwards undertaking similar duties themselves, when appointed to public asylums, or subsequently engaged in private practice. In every large institution for lunatics, the medical staff ought invariably to comprise resident pupils. Such a system works admirably in France, and would prove equally beneficial in the lunatic establishments of Great Britain.

### Montrose Royal Asylum.

The institution which now comes under review is the oldest public asylum for the insane throughout Scotland, having been founded seventy-three years ago, chiefly through the exertions of Mrs. Carnegie—a benevolent lady residing in the neighbourhood. When first opened, and during many years afterwards, it was the admiration of the surrounding country, the lunatic inmates being there taken care of and treated quite differently, compared with the system to which they had heretofore been subjected. Consequently, this asylum became almost a show-place, like Bethlem Hospital of the olden times. Whatever might be now thought of the treatment formerly pursued, great credit is certainly due to the humane individuals who patronized and supported its objects by subscriptions, as also official administration. However, like many things which have at last become antiquated, whilst science and civilization were advancing, even although in various respects this asylum has kept moving onward in the race of improvement—still, from original defective construction, and recent encroachments effected by commercial, money-making bodies in its immediate vicinity, the Montrose institution must now take an inferior position, compared with several insane establishments more recently constructed in Scotland.

At the period of its foundation, the asylum was situated in an open and airy portion of the "links" adjoining the town, then unencumbered by neighbouring houses. Now, the building is almost surrounded. On one side a new dock, often full of vessels, has been constructed. On another, a ship-building yard is established opposite, which nearly touches the dormitories occupied by female patients: and through the windows of which they can be easily seen by carpenters, workmen, or others; and *vice versâ*. Again, on the third side a railway station completes the obstructions. The asylum garden being also of limited extent, and having no adjoining fields, wherein the inmates might be employed, or obtain recreation unobserved, or, at least, be not interfered with by the passing public, constitute very objectionable features. To remedy such great defects, two farms, at a little distance, have very properly been leased by the official authorities; but, although these adjuncts comprise thirteen acres, they are not sufficient for every purpose.

When perambulating the different departments of this institution, the thought struck my mind that an examination of the numerous additions and improvements made since its foundation, would furnish a good history of the successive stages which have characterized the varied treatment of insanity in this country. Thus the old, dark, and badly-ventilated cells, considered so useful in 1780, —the confined sleeping apartments of a later period,—the alcoved dormitories constructed in the early part of the current century, —the more airy rooms built about 20 years ago,—and lastly, the cross-windowed, thoroughly-ventilated, as also exceedingly cheerful dormitory completed only very recently, which seemed really of a superior description, would each supply most instructive illustrations of the several epochs, as also of the modes of management pursued respectively.

An impartial observer need, therefore, only examine these several localities, in order to ascertain the prevailing opinions then entertained regarding lunatics, as well by medical men as philanthropists.

Although recently much has been accomplished to ameliorate defects, often inherent to all ancient constructions, the executive now deem it unseasonable to do more in this respect, than they reckon absolutely indispensable for the immediate health and comfort of patients: particularly, when suggestions for erecting a new asylum are under consideration. Several improvements of importance have nevertheless been completed. A large room has been divided into two apartments, to each of which a small gallery was also added for exercise, or the temporary seclusion of irritable inmates. Another spacious out-room has been also appropriated to female paupers. The alterations now specified have greatly contributed towards improving the classification, and considerably to diminish any over-crowding in the asylum, which was being the case recently, owing to an increased demand for admission. A new dwelling in the garden, intended for Dr. Gilchrist, the medical superintendent's, residence, having been liberally given up by that officer, to relieve the pressure of applicants, a large portion of it has now been fitted up temporarily for higher class females, whereby increased accommodation is afforded. The attendants upon patients have lately also been considerably augmented in number; so that at present, there is one attendant to about every fifteen lunatics. Still, in consequence of the imperfect structural arrangements belonging to an old building, and the absence of all mechanical appliances to lighten manual labour, these individuals are even yet unable to perform the same duties often accomplished in other institutions, where similar defects do not prevail. The ventilation of the older buildings, and especially the ground-floor cells, is very defective: whilst sufficient day and work rooms for inmates do not exist; the chief object, until of late, influencing the management having appeared, rather to augment the number of beds for receiving new patients, irrespective almost of any other consideration.

The dormitories throughout seemed clean, although sometimes too crowded with beds. The patients generally appeared quiet and orderly ; the resident officers having done everything they could to ameliorate their condition, in spite of irremediable deficient constructions and over-crowding. On the day of my visit, the total insane residents amounted to 229 : of whom 96 were males, and 133 females. Amongst these, however, 42 were classed as private patients, each paying from 25*l.* to 100*l.* per annum. The epileptics comprised 9 males and 8 females : whilst, 13 of the former and 20 of the latter sex were reported dirty persons. Besides which, it should be mentioned, as many recent cases of insanity, especially insane females, had been of late admitted, the institution, consequently, had thus become less of an asylum, and more like an hospital, than previously. In the dark cells, 9 females and 2 males were then confined: most of these cases being exceedingly violent, particularly the female lunatics, some of whom had so torn their clothes as to be nearly naked, and laboured under a high state of excitement. One female also occupied a kind of box bed, but padded throughout, and having a kind of net-work on the top, to prevent her getting out of this enclosure. Such contrivance was employed, because the party would neither lie quietly, in an ordinary bed, nor allow the clothes to remain on her person. Two days afterwards, when I again visited this asylum, along with my learned friend, Mr. Logan, Sheriff of the County, and who on that day made an efficient inspection of the whole establishment, the female above alluded to was sitting among other patients as usual, being then much more composed. Besides this fact, it is also satisfactory to report that, several of the other female inmates, secluded in dark cells only two days before, were now at liberty : their fits of excitement having passed like a transient storm, and thereby leaving the sufferers perfectly tranquil.

Notwithstanding the statements just made, and although it is admitted, according to the last annual report, that—

"Restraint is occasionally not only necessary, but proper, we have not been called upon to apply it in any instance during the year. Only one case has presented any difficulty,—the case of a self-mutilator, a young female labouring under chronic mania, resulting from intense hereditary predisposition. In three or four places, on the hands and arms, she had made large and even dangerous wounds, tearing out the soft parts with all the ferocity of a tiger. The determination had existed for some days, and, as may be supposed, the strictest watching was useless. I had already made up my mind to the necessity of its application, but, while meditating on the best form, I was gratified by the diminution, ultimately by the entire cessation, of the morbid propensity.

"Several cases, as formerly, have been brought to the house under restraint, all of which have had their liberty granted at once, without difficulty or danger. One of them—a married female—deserves notice. A strong piece of wood was inserted bit-wise between the teeth, and firmly secured by a strong cord tied behind the neck. The reason assigned was that the patient had severely bitten her tongue. This instrument of torture was at once removed, with great relief to the sufferer. On its removal, both angles of the mouth were ascertained to be in a state of ulceration, from the pressure of the wood, and the tongue presented a fœtid and sloughing mass to the depth of an inch. The patient was in so anæmic and exhausted a condition as to render recovery almost hopeless. She has, however, done well, but still labours under a certain degree of mental depression, and some impediment of speech, from the loss of so large a portion of the 'unruly member.' It is sadly interesting to note that each of these patients has a brother in the house."

Similar examples of cruel physical coercion towards excited lunatics were formerly not uncommon. Now, these are much less frequent; thanks to recent discussions in reference to non-restraint, and the diffusion of more correct notions amongst the community at large, respecting mental alienation.

To show the remarks made, in previous paragraphs, regarding the irremediable defects of the present buildings, are not overdrawn or unfounded, I would here append the observations made by the present able medical superintendent, Dr. Gilchrist, who says—

"It will be readily admitted that, in all asylums, the basement stories are less perfect in their sanitary conditions than the upper, partly from their position and structure, more especially from the class of patients who inhabit them. When it is remembered that Montrose Asylum is the oldest in Scotland, and that the original cells (built in 1781) are still occupied by the patients, it will readily be granted, without farther proof, that the house is in a less satisfactory condition than most other establishments, as to these cells at least. Here, as everywhere, a number of patients have acquired dirty habits, due not to the disease under which they labour, but to neglect in its earlier stages, in most cases before they have been placed under appropriate treatment. Truth compels us to state that the proportion of such cases is much greater here than usual. This is due to several causes, some of which are happily now removed, but others of them are irremovable, depending partly on the imperfect structure of an old house, and partly on the very partial supply—in some cases entire absence,—of the means necessary to the improvement, removal, or prevention of such habits."

Such being the acknowledged defective condition of this now venerable establishment, the resolution very recently passed by its executive, to construct another institution in the vicinity, is highly commendable. A piece of ground, about thirty acres, has already been purchased for that purpose: the situation chosen being an elevation, with a southern exposure. It is airy, salubrious, and lying a few miles north of Montrose, will not be obstructed by any neighbouring buildings. Altogether, the selection made of Sunnyside—the truly descriptive name this locality now bears—seems judicious; and doubtless the new asylum will justify the anticipations formed of its future capabilities.

Throughout last year, 91 new patients were admitted; of whom 39 were male, and 52 female lunatics. The cures amounted to 37, the sexes being nearly equal—seeing 18 male to 19 female patients were discharged convalescent; which makes the proportion upwards of 40 per cent. upon the admissions. On the other hand, the deaths were 21—composed of 11 males and 10 females; which, therefore, gives a ratio of 23 per hundred, if also calculated according to the numbers admitted. Amongst the deaths reported, 4 were cases of general paralysis, 4 arose from epileptic seizures, 3 from chronic bronchitis, 2 resulted from apoplexy, 2 were caused by maniacal exhaustion: 3 males, respectively, died of consumption, diarrhœa, and diabetes; whilst 3 females were also cut off by broncho-pneumonia, cardiac disease, and fatal syncope—the largest mortality being recorded in the month of December, when four patients died; whereas, the fewest occurred during mild weather. In reference to the type of mental alienation, with which the 21 cases terminating fatally were affected: it may be interesting to mention that, 4 laboured under general paralysis, 4 epileptic mania, 4 chronic mania, 2 monomania, 2 melancholia, 2 dementia, one acute mania, and another senile insanity; the last being an example of suicidal melancholia.

Occupying the inmates constitutes a prominent feature at this establishment, like most well-regulated asylums. Consequently, as the report states, usually there have been employed of males, two as tailors, as joiners two or three, as shoemakers one or two, as net-makers four to six, as gardeners six to eight, as field labourers ten to fifteen, as oakum-pickers fifteen to twenty, and others occupied in the galleries, laundry, at the pump, pig feeding, from five to ten—thus fifty to sixty were employed, exclusive of the higher class private patients. Thirty or forty remaining unemployed. The greater number of the pauper patients, especially from the immediate neighbourhood, being mill-workers, this diminished the proportion of those trained in handicrafts, and rendered them less ready to engage in such occupations. Out-door labourers appeared still fewer than could be wished: but the smallness of the garden, and the distance and exposure of the fields, made it almost impossible to employ a greater number. Again, regarding females, twenty to thirty were engaged in sewing, five to ten in knitting, some in crochet and other fancy work, with about twenty in the galleries, kitchen, and laundry,—giving nearly fifty in a hundred. Attempts towards increasing the workers on the female side, have hitherto not been so successful as on the male. An arrangement, however, has just been completed, by the removal of the higher class females to the new building, which provides a work-room ; and, as an additional qualified attendant has been engaged to superintend, one of whose special duties will be to induce and encourage patients to engage in such occupations, better results are anticipated in future.

Recreating, as also instructing the insane patients, is justly not overlooked ; on the contrary, such appliances in their treatment seem liberally supplied. Thus, lectures, concerts, and other sources of amusement and instruction in the town are visited, whenever an opportunity occurs, and to which convalescent paupers, as also private patients, have access. Lecturers, vocalists, bands of music, and so forth, are likewise frequently secured, wherever possible. Besides the above, there is a weekly dance, which forms a great centre of attraction, in addition to other amusements, constituting often an important item of moral improvement. Skittles, quoits, bowls, and drilling, with other out-door games, being often arranged during summer. Pic-nics, excursions to the country, and visiting various scenes of interest in the neighbourhood—highly deserving notice, on account of their natural beauty and historical relations—have often been participated in by numerous pleasure-seeking parties of inmates, who could properly appreciate or benefit by such privileges.

Reading and intellectual culture, whenever applicable in such a community, are further far from being neglected. Newspapers and serials being supplied, to satisfy this appetite—natural even in lunatics. Besides the literary stores of this institution, which furnishes its quota of amusement and instruction, portions of the more fastidious reading inmates are supplied with books from the public library in town. Attendance on religious services also constitutes an important feature ; and recently, a large addition has been made to the number of patients attending chapel, many being even inmates who, formerly, would have been deemed unfit to engage in such duties. A considerable portion of the community, male and female, both of pauper and private patients, occasionally attend the public churches of Montrose, with evident advantage.

Recently, the physical health of most residents has been good, and, on the whole, satisfactory. Unlike the former season, cholera has not prevailed in this establishment since that period ; but it seems worth mentioning, on the other hand, that in the beginning of last February, erysipelas broke out, five cases having occurred in four weeks ; whilst lately, an epidemic of apoplexy made its appearance: four instances of that disease having occurred during five weeks; three being females in advanced life, and whose insanity had been of long standing. Of the above, two died after a few days' illness: whilst the third, it was reported, has now nearly reached her former condition, viz., chronic mania, with periodical excitement. Very few patients were sick in bed, the day I visited the asylum: and, in this respect, there seemed nothing novel or worthy of remarking.

Notwithstanding the average insane patients, irrespective of attendants, varied last year from 215 to 220, and the number amounts now to 229, only one resident medical officer is attached to this institution—viz., Dr. Gilchrist, who is likewise superintendent. At the Glasgow Asylum, with 381 lunatics, there are three medical attendants, as already stated, which gives one to every 127 patients. Consequently at Montrose, there ought to be at least one resident assistant also, seeing the physician has by by far too many duties now to perform. Not only has that officer to undertake occupations personally, which may be called manual, and of minor importance, but he must likewise attend to and supervise the entire establishment. Further, as many inmates are ladies and gentlemen, who become often more difficult to manage than pauper patients, they are in a higher degree exacting as to the attendance required; besides which, seeing such cases contribute greatly towards the profit of this institution, its present medical staff is certainly too limited. In fact, this defect is even more prominent than at Dundee, where, although the asylum contains fewer lunatics, there is also an attending physician, who shares the responsibility with Dr. Wingett. Here, moreover, irrespective of the patients being in greater number, the medical superintendent has no professional assistant. Hence, if absent for recreation or otherwise, only for short periods, which occasionally must occur, as that gentleman is not a prisoner, proper aid may not be readily procured, should any emergency supervene.

Some months ago, a second medical officer was voted by the Board of Management; but difficulties respecting the appointment having arisen, and as only a very paltry salary was proposed, hitherto no result has followed this proposition. Indeed, it is said, several parties did not think the measure absolutely necessary, since they considered four or five hours per day were quite as much as any person could possibly spend on the patients under treatment. If such opinions really existed, these authorities would almost seem to have an impression that, the medical superintendent of an asylum has really no other functions to undertake, beyond those of a mere prescriptionist. Moral management must then constitute no part of the conception entertained, regarding the great utility of possessing an adequate professional staff, in extensive institutions for the insane. Notions of that description are most erroneous; and wherever it should be still supposed an additional medical officer is merely wanted, for the purpose of preparing medicines, dressing sores, or to perform minor operations, and be engaged only in inferior employments, such an idea is altogether a mistake. Consequently, both at Dundee, but especially in the asylum now under review, there ought to be, at least, two resident medical attendants.

Before concluding these remarks, I must however add—indeed, my now doing so is only an act of simple justice—that Dr. Gilchrist performs his varied yet multifarious duties most zealously, even although these have been, of late, greatly augmented, as well from the introduction of many new attendants—at first but imperfectly acquainted with their several occupations—as also on account of the large accession of new patients. Besides the above important considerations, it should further be noticed, in consequence of frequent and sometimes important changes of internal arrangements, Dr. Gilchrist's labours have lately been materially increased; whereby the onerous and other functions taxing the energies of that officer did not invariably prove so smooth in their course as could have been wished, or the marked assiduity displayed fairly entitled him to anticipate.

(*To be continued.*)

---

